# Supplementary material for: Genetically synthesized supergain broadband wire-bundle antenna
Source: Commun Eng. 2024 Jul 23;3:101. doi: 10.1038/s44172-024-00235-y (PMC11263590; doi:10.1038/s44172-024-00235-y)
Supplement: Supplementary file 2 — Supplementary Information [file 44172_2024_235_MOESM2_ESM.pdf]

Genetically Synthesized Supergain Broadband Wire-Bundle Antenna

Dmytro Vovchuk[1,=,*], Gilad Uziel[1,=], Andrey Machnev[1], Jurgis Porins[2], Vjaceslavs Bobrovs[2], and Pavel Ginzburg[1]

1. School of Electrical Engineering, Tel Aviv University, Ramat Aviv, Tel Aviv, 69978, Israel
2. Institute of Photonics, Electronics and Telecommunications, Riga Technical University, Riga, LV-1048, Latvia

*Corresponding author: dimavovchuk@gmail.com

[=] These authors contributed equally

Supplementary Note 1

Other antenna layouts

Supplementary Figure 1 (a-f) presents the top and perspective views of three antennas (AUT 1-3), which were obtained with the genetic algorithm. The AUT1 is the main antenna that is comprehensively addressed in the main text. Two others (AUT2 and AUT3) are additional outputs of the algorithm, possessing similar characteristics – directivity and gain. Numerically estimated properties of the antennas appear in Supplementary Figure 1 (g-h). The radiation pattern properties are governed by multipole expansions, which vary between the antennas. Supplementary Figure 1 (j) shows the expansion to AUT3, which may be compared to the expansion for AUT1 in the main text.

Antennas efficiencies appear in Supplementary Figure 1 (i). It is clearly seen that the efficiencies of AUT1, 2, and 3 have 8.4, 11.76, and 5.96% of fractional bandwidth BW, respectively. The overall efficiencies in the band approach 0.9-1.

Considering the radii of the spheres, enclosing the antennas (14.08, 16.58, and 13.7 mm, respectively), the comparison with Chu-Harrington and Geyi limits was performed. AUT 2 overcomes both limits, while AUT 3 is only Geyi limit (Supplementary Figure 1 (k-l)). AUT 1 has the best performance according to this figure of merit and, thus, was chosen for further experimental studies.

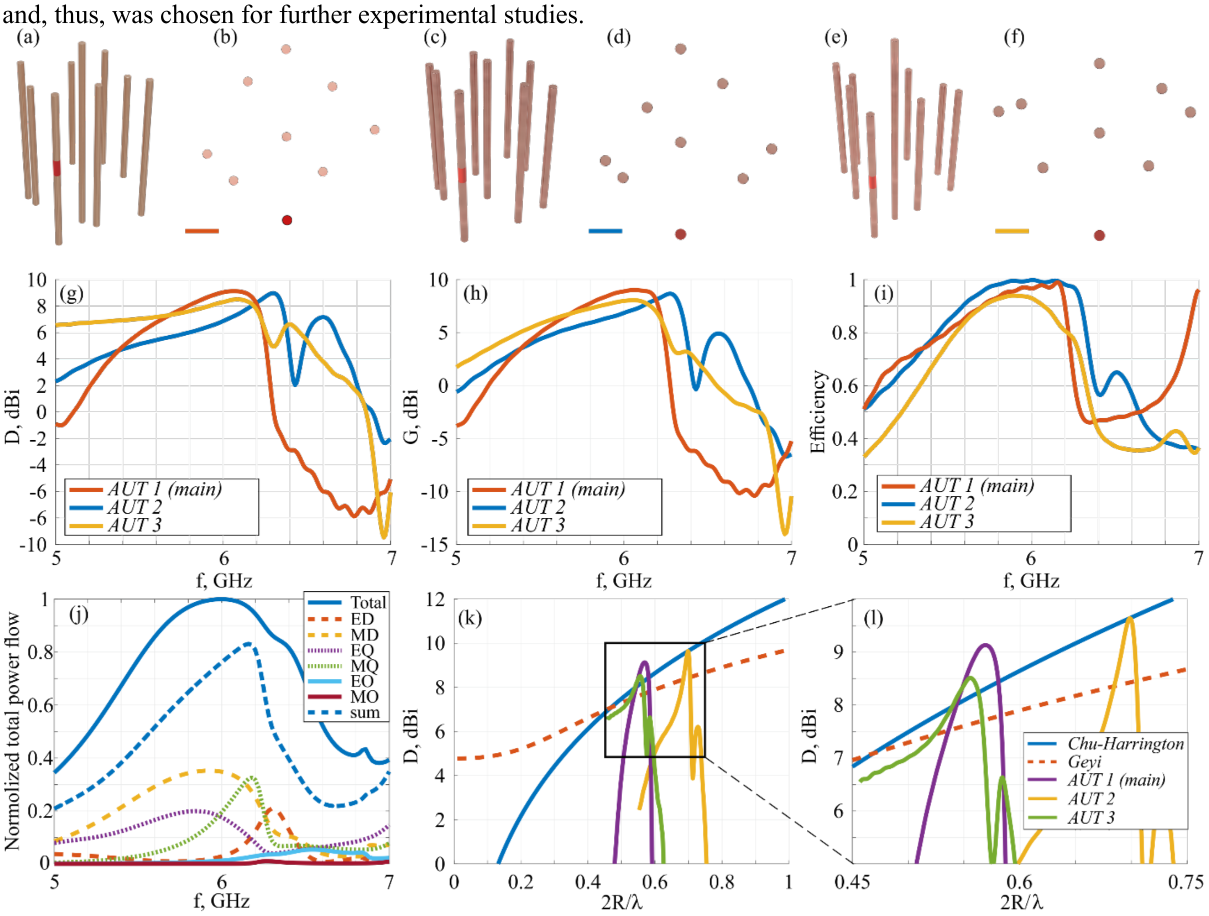

Supplementary Figure 1. Other antenna layouts and their characteristics assessment. (a-f) The perspective and top views of AUTs. (g) Directivity, (h) gain, and (i) efficiency of the antennas. (j) The multipole expansion of AUT 3 radiation pattern. (k) - the antennas' directivities, assessed versus Chu-Harrington and Geyi limits. (l) Zoom in to panel (k).
